# Successful Treatment of Noninflammatory CaHA Nodules Using Focused Mechanical Vibration

**DOI:** 10.1093/asjof/ojae018

**Published:** 2024-03-21

**Authors:** Alec D McCarthy, Shoham Berkowitz, William Gregory Chernoff

## Abstract

Noninflammatory nodules arising from the injection of biostimulatory fillers persist as an unwanted complication. Pathologically, noninflammatory nodules may arise from superficial injection, accidental boluses, or incorrect concentration of microparticles contained within the filler. This case report introduces a method for reversing calcium hydroxylapatite (CaHA) using focused mechanical vibration. An in situ hyperdilution was created by injecting saline into the nodule core to prepare it for resuspension. Topical microneedling was subsequently applied to generate vibrations, aiming to disperse the accumulated CaHA particles. The outcome demonstrated a significant reduction in the size and visibility of the nodule. This combined saline-microneedling approach offers a potential noninvasive, nonpharmacologic solution for managing superficial CaHA nodules.

**Level of Evidence: 5:**

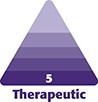

Radiesse (CaHA-CMC) is a regenerative biostimulator composed of 30% w/v calcium hydroxylapatite (CaHA) and 70% w/v carboxymethylcellulose (CMC) gel that has grown in popularity over recent years.^1^ CaHA-CMC has garnered popularity for its unique dual benefits: it both immediately fills and volumizes soft tissue and also stimulates the production of key extracellular matrix (ECM) proteins, including Collagen 1, Collagen 3, elastin, and proteoglycans, resulting in regeneration of the ECM.^2-5^ A unique attribute of CaHA-CMC is the ability to adjust its rheology through dilution, allowing for a more tailored approach to meet diverse patient needs and aesthetic goals.^6^

As with all particle-containing injectables, complications may occasionally arise. Noninflammatory nodules, primarily attributed to the focal accumulation of CaHA microspheres, can present as palpable and sometimes visible nodules, which may become a cosmetic concern. The pathogenesis of these nodules is distinct from inflammatory reactions and is fundamentally rooted in the clustering of these microspheres rather than an immune-mediated response.^7,8^ Poor dispersion in situ, caused by accidental superficial placement, accidental bolusing, injecting large volumes of highly concentrated product, migration, and/or a poor injection pattern, may contribute to noninflammatory nodule formation.^9^

In the past, dispersion with aqueous solution, thermal mechanical abrasion, and a variety of pharmacological interventions have been deployed as methods to reverse nodules with a wide range of efficacy.^8,10,11^ Recently, an algorithmic approach for reversing CaHA focal accumulations was presented, which introduced the idea of mechanically assisted dispersion.^12^ Based on the use of focused mechanical vibration (FMV) to reverse plaques of other origins, we sought to deploy FMV in conjunction with aqueous dispersion to rapidly reverse the noninflammatory nodules based on the following rationale: (1) the initial application of warm compress would facilitate increased perfusion in the area, (2) directly dispersing the nodule with an aqueous solution would loosen the particles and provide a medium for further resuspension, and (3) the FMVs would shake the particles apart and facilitate in situ homogenization (Figure 1).

**Figure 1.**
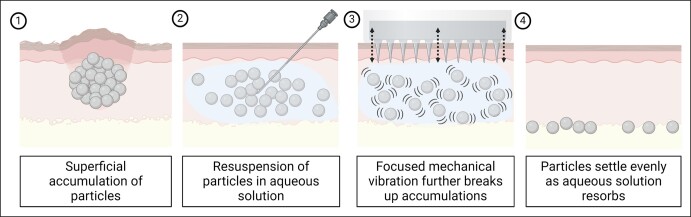
Proposed mechanism of action for focused mechanical vibration to resolve CaHA nodules.

## CASE REPORT

**Figure 2.**
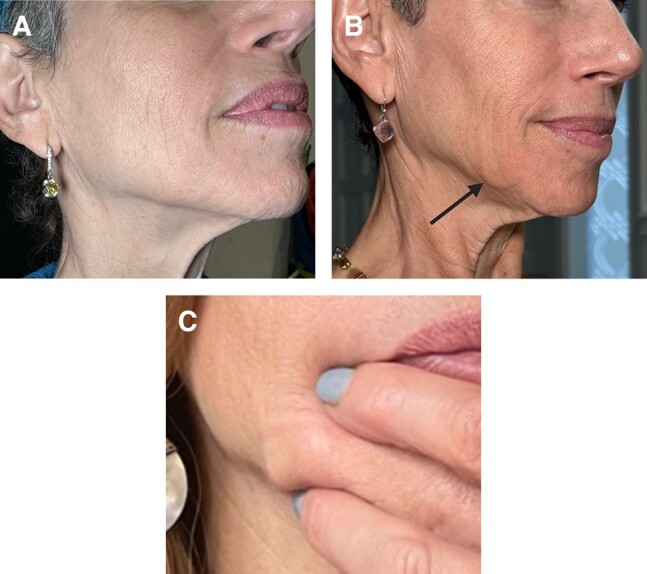
(A) Pre-treatment photos show no nodules in the jawline. (B) Post-treatment nodule visible at rest which is easily visualized with (C) manual palpation.

**Figure 3.**
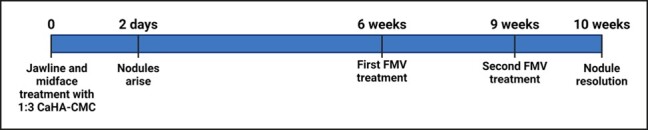
Treatment timeline starting at 0 (treatment day) and ending 10 weeks after the initial treatment.

A patient was treated with subdermal hypderdilute (1:3) CaHA-CMC in the jawline and midface with a cannula and presented 6 weeks posttreatment with bilateral noninflammatory nodules in the jowls that were not present prior to treatment and appeared around 48 h after the initial treatment, as visualized in her pre- and posttreatment photographs (Figure 2A, B). The patient could easily palpate the nodules, which were visible at rest, and they were approximated to be 0.4 to 0.5 mL in volume (Figure 2C). No edema, pain, redness, or heat accompanied the nodules. While not painful, the nodules negatively affected the aesthetics of the patient and accentuated her jowling. The patient opted for reversal of the nodules when presented with “wait and watch” or treatment options.^13^ Thus, 6 weeks after CaHA-CMC treatment, the patient was treated with FMV, was treated a second time with FMV 9 weeks after CaHA-CMC treatment, and reported resolution at 10 weeks post-CaHA-CMC treatment (Figure 3).

### Reversal Protocol

Dispersion alone has been shown to reduce the size of CaHA nodules by up to 30%, although it was hypothesized that the rapid neocollagenesis and tissue integration may inhibit complete resolution in stubborn nodules.^1^ Ablative technologies have also shown efficacy in mechanically disrupting nodules.^8,14^ We sought to draw from both the dispersion and FMV mechanisms of action.

Thus, this protocol deployed began by applying warm compress to the nodules with a heating pad for 5 min. Next, 0.9 mL of solution (0.7 mL saline, 0.2 mL lidocaine) was injected directly into the nodules with a needle, effectively creating an in situ dilution for the CaHA particles to disperse into. A mechanized microneedle (Dr Pen Ultima M8, Dr Pen, Phoenix, AZ) equipped with a sterile 36-pin cartridge was then applied directly over and around the nodules in 3 continuous rolling directions for 5 min per nodule at the highest speed setting (around 15,000 RPM) and at a depth of 1.0 mm.

**Figure 4.**
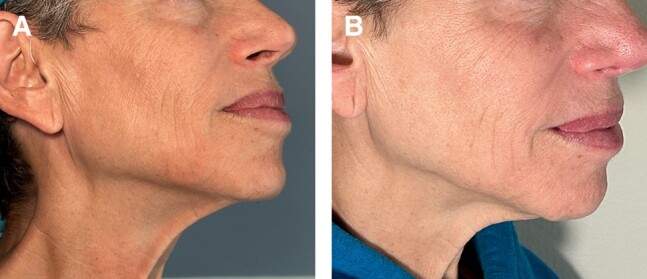
Results after treatment. (A) Results 1 week after the first round of FMV treatment. (B) Result 2 weeks after the second round of FMV.

Immediately after the first session of microneedling, the nodules were reduced by approximately 30%. Two and 7 days after the initial treatment, the nodules were reduced by approximately 50% and 70%, respectively (Figure 4A). A second session following the same protocol was carried out 3 weeks later with 80% resolution immediately after the second round of FMV and near full resolution 1 week later (Figure 4B). The patient has not had any recurrent nodules after the second nodule reversal treatment and did not report any complications related to the reversal protocol and only reported mild erythema persisting for around 2 h post-FMV. The 2-month delay between reversal protocols was due to the patient living out of state.

## DISCUSSION

We report, for the first time, FMV for the reversal of noninflammatory CaHA-CMC nodules. The immediate improvement in nodules is likely attributed to the immediate dispersion effect enhanced by the focal vibration. Improvement over the next several days is attributed to the reduction in edema resulting from the microneedling and the gradual resorption of the aqueous solution.^15,16^ The success of this mechanical intervention relies on the dual mechanisms of action: aqueous dispersion from injecting an aqueous solution and mechanical vibration imparted by the microneedling device. Depending on how long the nodules have persisted, different aqueous solutions may be optimal choices and are discussed below.

Saline, due to its safety, biocompatibility, and cost-effectiveness, is often the first choice as an aqueous diluent. Its isotonic nature ensures minimal tissue irritation and can be readily mixed with lidocaine. Collagenase may be considered in cases where a fibrous capsule surrounds the nodule that essentially anchors the CaHA particles together. The collagenase would enzymatically degrade the collagen holding the particles in place, allowing for resuspension.^17^ Collagenase may be necessary for persistent nodules that have remained untreated for a long period of time. While primarily used to dissolve hyaluronic acid fillers, hyaluronidase might enhance the dispersion of CaHA particles by breaking down any surrounding endogenous hyaluronic acid within the ECM.^18,19^ Neither collagenase nor hyaluronidase, however, would have any effect on the CaHA nor CMC components of the CaHA-CMC nodules.

**Figure 5.**
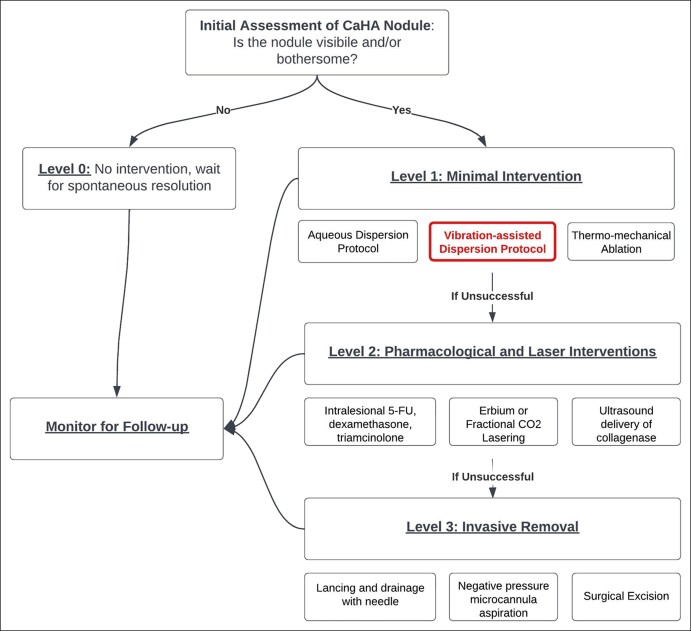
An algorithm proposed by McCarthy et al., of which FMV falls under Level 1 interventions. Reprinted with permission from McCarthy et al., *Aesthetic Surg J*. 2024;sjae031. doi: 10.1093/asj/sjae031. Copyright 2024 Oxford University Press.

The authors, in this case, used microneedling to induce FMV, though it is hypothesized that a variety of devices could theoretically be used for such cases. Microneedling devices are generally low cost and abundant in aesthetic offices and have minimal risk of complications, nor do they significantly affect the underlying tissue. Still, adverse events such as erythema, risk of infection, pain, and dyspigmentation associated with microneedling should be considered and discussed with the patient. Other devices to induce FMW may be cavitation ultrasound, percussive devices, or piezoelectric devices. Namely, any noninvasive or minimally invasive tool that allows for focused regional vibration may aid in dispersion. As such, this nodule dispersion methodology would fall into the algorithm proposed by McCarthy et al as a Level 1 intervention—minimally invasive vibration-assisted dispersion (Figure 5).^10^

Of note, several limitations exist in this case report. First, the authors had no previous guidelines on needle depth or duration for the microneedling process, and instead, used their clinical judgment and tactile evaluation throughout the procedure to palpate the nodules or lack thereof. These parameters could be further optimized in future studies. Second, this is a singular case that lacks histological evaluation. Therefore, it is difficult to attribute the dispersion of the nodules to the FMV or the aqueous dispersion. Finally, the authors only explored the use of microneedles as the device to induce FMV, and it is unclear if other devices capable of inducing local tissue vibration may perform better for this procedure.

## CONCLUSION

The utilization of FMV in conjunction with aqueous dispersion presents a promising strategy for the rapid reversal of noninflammatory nodules resulting from CaHA-CMC injections. The rationale for this treatment is based on creating a dilution medium for resuspension of CaHA microspheres and near-ultrasonic vibration to facilitate resuspension with microneedling. The immediate and sustained reduction in nodule size posttreatment, as observed in this case report, underscores the efficacy of this approach. Saline is suggested as the dispersion agent in this case, although diluents, such as collagenase and hyaluronidase, may offer advantages for resolving more stubborn or long-standing nodules. Although microneedling was used for vibration, it is hypothesized that various devices could be used to induce FMV. This novel intervention effectively resolved bilateral CaHA nodules with no invasive procedures nor any pharmacologics.
